# *Tsc2* Haploinsufficiency Has Limited Effects on Fetal Brain Cytokine Levels during Gestational Immune Activation

**DOI:** 10.1155/2014/761279

**Published:** 2014-07-09

**Authors:** Dan Ehninger

**Affiliations:** German Center for Neurodegenerative Diseases (DZNE), Ludwig-Erhard-Allee 2, 53175 Bonn, Germany

## Abstract

Dysregulated TSC/mTOR signaling may play a pathogenetic role in forms of syndromic autism, such as autism associated with tuberous sclerosis, a genetic disorder caused by heterozygous *TSC1* or *TSC2* mutations. Environmental risk factors, such as gestational viral infections, may, in some cases, also contribute to the pathogenesis of autism and related neuropsychiatric disorders. We have recently found that a heterozygous *Tsc2* mutation and the poly I:C model of maternal immune activation (MIA) interactively perturb fetal development and adult social behavior in mice, suggesting that these factors converge on shared pathways. TSC/mTOR signaling plays an important role in the modulation of immune responses, raising the possibility that the damage caused by MIA was greater in *Tsc2^+/−^* than in wildtype fetuses because of an exacerbated immune response in the mutants. Here, cytokine antibody arrays were employed to measure relative cytokine abundances in the fetal brain and the placenta during MIA. Cytokines were induced by gestational poly I:C but there was no obvious modulatory effect of *Tsc2* haploinsufficiency. The data indicate that cytokine exposure during MIA is comparable in *Tsc2* haploinsufficient and wildtype control fetuses, suggesting that downstream molecular and cellular processes may account for the interactive effects of *Tsc2* haploinsufficiency and MIA.

## 1. Introduction

Dysregulated mTOR signaling and altered protein synthesis are emerging as common themes in the biology of autism [1–3]. In brief, heterozygous mutations in the* TSC1* or* TSC2* gene cause tuberous sclerosis, a multisystem disorder, which is associated with autism in 20–60% of cases [4–6]. Additionally, mutations in the genes encoding for the upstream regulators PTEN and NF1, as well as in the downstream effector eIF4E, have been linked to autism [7–9]. Moreover, fragile X syndrome, another single gene disorder, in which the regulation of mRNA translation is perturbed [1], is associated with autism [11]. These data collectively highlight the mTOR pathway and protein synthesis as one important theme in the pathogenesis of autism-related disorders.

In addition to genetic factors, environmental risk factors may also contribute to the pathogenesis of autism spectrum disorders. At least some prenatal viral infections elevate the risk for neuropsychiatric disorders (such as autism spectrum disorders and schizophrenia) in the offspring [10, 12–16]. Moreover, there is a growing literature suggesting the presence of inflammatory or immunological changes in the brains of at least a subset of individuals affected by autism [17–21], suggesting that immune activation/inflammatory processes may play a role (primary and/or secondary) in the pathogenesis of some cases of autism.

Prenatal viral infections have been modeled in mice by injection of poly I:C [19], a synthetic double-stranded RNA that elicits an immune response via activation of toll-like receptor 3. Gestational poly I:C injections in mice and rats elicit behavioral, neurochemical, and structural abnormalities in the developing offspring [19, 22, 23], which is consistent with the notion that maternal immune activation during gestational periods can alter proper neurodevelopment and, hence, results in behavioral consequences in the offspring generation.

Heterozygous* Tsc2* mutations and the poly I:C paradigm of maternal immune activation (MIA) showed significant interactive effects in mice, indicating a cooperative influence on gestational survival and postnatal behavioral traits [24]: fewer *Tsc*2^+/−^ pups than wildtype (WT) pups were born to poly I:C injected dams, while the number of *Tsc*2^+/−^ pups and WT pups born to vehicle-injected mothers did not differ. Moreover, grown-up mice exposed to both gestational poly I:C and* Tsc2* haploinsufficiency showed deficient social exploratory behavior, while this was not the case in the animals exposed to one of these factors alone.

Gestational immune activation may disrupt normal brain development at least in part because of crosstalk between cytokines and the developing CNS [19, 22, 25–27]. TSC/mTOR signaling regulates immunological and inflammatory processes [28, 29] and, accordingly, it is possible that an exacerbated immune response in the *Tsc*2 mutant fetus accounts for the more severe consequences of gestational poly I:C in the *Tsc*2^+/−^ background. To address this possibility, *Tsc*2^+/−^ fetuses and WT control fetuses were gestationally exposed to poly I:C or saline and cytokine responses were measured in the placenta, as well as the fetal *Tsc*2^+/−^ and WT control brain.

## 2. Materials and Methods

### 2.1. Mice

Mice were generated as previously described [24]. In brief, in order to generate *Tsc*2^+/−^ and WT offspring gestationally exposed to either poly I:C or saline, *Tsc*2^+/−^ male breeders (on a C57BL6/Ncrl genetic background) were mated with C57BL6/J wildtype females. After overnight mating, female breeders with a vaginal plug were designated as E0, single-housed, and left undisturbed, except for weekly cage change. At E12.5, pregnant females received a single intraperitoneal injection of 20 mg/kg poly I:C (Sigma) or vehicle (see below). Poly I:C is supplied as potassium salt and poly I:C constitutes 10% of the total weight of the salt. The dosage was based on the actual weight of poly I:C itself. Poly I:C was dissolved in 0.9% sterile saline and a volume of 10 *μ*L/g was injected. Local and federal regulations regarding animal welfare were followed.

### 2.2. Tissue Extraction

Two hours or 6.5 hours after poly I:C, pregnant females were anaesthetized with isoflurane and were decapitated. The uterus was extracted and briefly washed in ice-cold phosphate-buffered saline. Subsequently, fetal brains and placentas were isolated in fresh ice-cold phosphate-buffered saline under a dissection microscope. Tissue was flash frozen and stored at −80°C until processed for cytokine antibody arrays.

### 2.3. Cytokine Antibody Arrays

Cytokine antibody arrays (RayBio Mouse Cytokine Antibody Array 3 and RayBio Mouse Cytokine Antibody Array 2) were used according to the manufacturer's instructions. In brief, tissue was lysed in the cell lysis buffer provided after addition of proteinase inhibitors (Sigma, P8340), homogenized, and centrifuged at 5000 g for 10 min (4°C) and the supernatant was isolated. Protein concentrations were established with BCA assays. Next, the volume containing 125 *μ*g protein was taken from each sample and pooled by group (WT/saline, WT/poly I:C, *Tsc*2^+/−^/saline, and *Tsc*2^+/−^/poly I:C; materials of 4 animals from 4 different litters were pooled per group for each experiment; at the 6.5 h time point, only fetal brain from the following groups was analyzed: WT/saline, WT/poly I:C, and *Tsc*2^+/−^/poly I:C). Pooled lysates were then diluted at least 10-fold in 1x blocking buffer to a total volume of 2.5 mL. After a blocking step, array membranes were incubated in pooled lysates at 4°C overnight. Next, membranes were washed 3 times in 2 mL wash buffer I for 5 min each followed by washing steps in wash buffer II (2 × 5 min; room temperature). Array membranes were then incubated in biotin-conjugated antibody solutions for 2 h at room temperature followed by washing steps in wash buffer I (3 × 5 min) and II (2 × 5 min). Next, membranes were incubated in HRP-conjugated streptavidin (1 : 1000) at room temperature for 2 h. After washing (wash buffer I, 3 × 5 min; wash buffer II, 2 × 5 min), array membranes were incubated in ECL-plus and imaged on a Typhoon 9400 scanner. Image analyses were performed in ImageQuant 5.2. Array membranes contained a total of 62 or 32 different cytokine antibody probes (RayBio Mouse Cytokine Antibody Array 3 and RayBio Mouse Cytokine Antibody Array 2, resp.), in addition to positive controls and negative controls. Fluorescent intensities were measured for cytokine probes and positive and negative controls. After subtraction of background, data for cytokine probes were normalized to the average fluorescence intensity of the positive controls. All data are expressed as percentage of the corresponding value of the WT/saline group and are based on duplicate measurements for each cytokine probe (shown as mean +/− SEM).

### 2.4. Statistics

In order to compare relative cytokine abundances across genotypes and treatment groups, multifactorial ANOVAs were performed (with genotype and/or treatment as between-subjects factor and/or cytokine as within-subjects factor) as described in the main text.

## 3. Results

To evaluate if fetal* Tsc2* haploinsufficiency modifies gestational cytokine responses during a maternal immune activation paradigm [22], pregnant C57BL6/J female mice were subjected to a single poly I:C injection (20 mg/kg, i.p.) or saline control at E12.5. Fetal brains and placental tissue were extracted 2 h or 6.5 h after the injection and the relative cytokine abundance in these tissues was assessed using cytokine antibody arrays.

**Figure 1:**
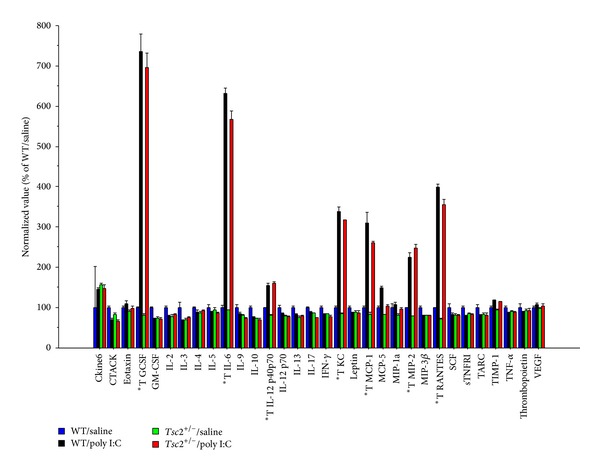
The graph shows relative cytokine abundances in placental tissue lysates at 6.5 h after poly I:C injection as measured by cytokine antibody array. *T denotes individual cytokines with main effect of treatment at *P* < 0.01 and a fold change > 1.5. Plotted are means +/− SEM.

In placental tissue (6.5 h after poly I:C), poly I:C caused a robust increase of several cytokines, including the proinflammatory cytokine IL-6 (Figure 1). An overall comparison of cytokine profiles by three-way ANOVA with genotype and treatment as between-subjects factors and cytokine as within-subjects factor yielded a significant main effect of poly I:C treatment (*P* = 0.0003). Additional analyses on the level of individual cytokines (using two-way ANOVAs with genotype and treatment as between-subjects factors) showed clear increases of G-CSF, IL-6, IL12 p40/p70, KC, MCP-1, MIP-2, and RANTES in the placental samples of poly I:C treated animals (Figure 1). Poly I:C-induced placental cytokine levels were similar in *Tsc*2^+/−^ and WT samples (Figure 1).

**Figure 2:**
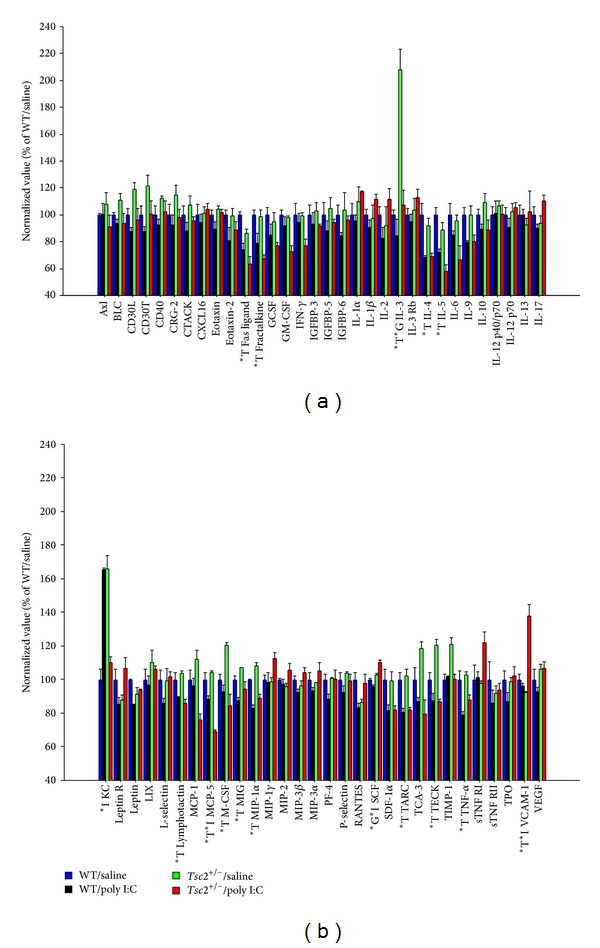
(a) and (b) The figure shows relative cytokine abundances as measured by cytokine antibody arrays in fetal brain at 2 h after gestational poly I:C. Individual cytokines with a main effect of treatment (*T; *P* < 0.01), genotype (*G; *P* < 0.01), or a genotype x treatment interaction (*I; *P* < 0.01) are highlighted. Shown are means +/− SEM.

Poly I:C effects on cytokines in fetal whole brain extracts were more moderate than those seen in the placenta: At 2 h after poly I:C, the levels of several cytokines appeared to be decreased in the treated group compared to saline controls (Figures 2(a) and 2(b)). An overall comparison of cytokine profiles (by three-way ANOVA with genotype and treatment as between-subjects factors and cytokine as within-subjects factor) showed a nonsignificant trend towards an effect of poly I:C (*P* = 0.09), while there was no effect of genotype (*P* = 0.34) and no genotype x treatment interaction (*P* = 0.95). Exploratory statistical analyses focusing on individual cytokines (using two-way ANOVAs with genotype and treatment as between-subjects factors) revealed a number of molecules with significantly altered abundances in the poly I:C groups (Figures 2(a) and 2(b)), such as Fas ligand, fractalkine, IL-3, IL-4, IL-5, lymphotactin, MCP-5, M-CSF, MIG, MIP-1alpha, TARC, TECK, TNF-alpha, and VCAM-1. These analyses, however, showed few effects of* Tsc2* haploinsufficiency on fetal cytokine levels (Figures 2(a) and 2(b)).

**Figure 3:**
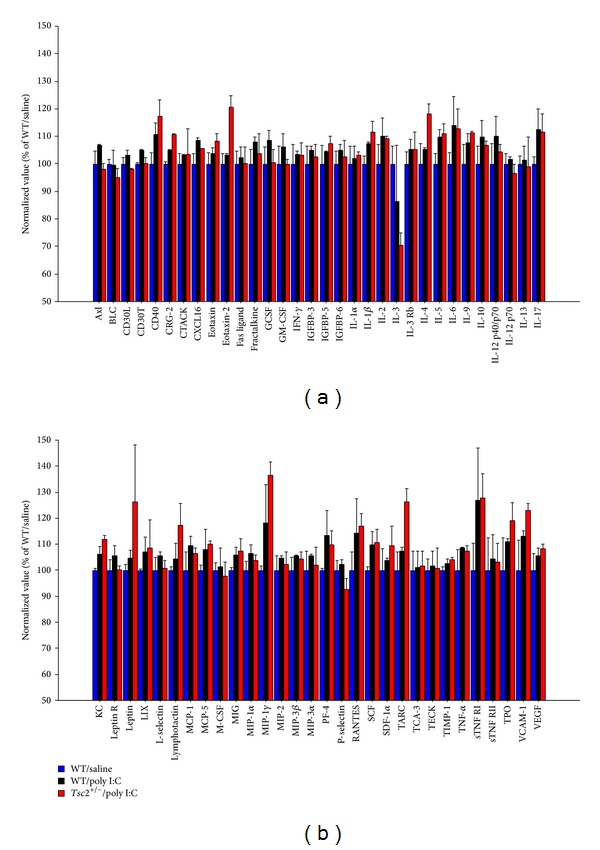
(a) and (b) This graph shows relative cytokine abundances as measured by cytokine antibody arrays in fetal brain at 6.5 h after gestational poly I:C. Shown are means +/− SEM.

At 6.5 h after poly I:C, cytokines appeared to be, overall, modestly elevated in poly I:C fetal brains relative to controls, although this finding did not reach statistical significance (Figures 3(a) and 3(b); two-way ANOVA, WT/saline versus poly I:C, treatment as between-subjects factor and cytokine as within-subjects factor: effect of treatment, *P* = 0.09).* Tsc2* haploinsufficiency did not seem to modulate the cytokine response induced by gestational poly I:C in any obvious way (Figures 3(a) and 3(b); two-way ANOVA, WT/poly I:C versus *Tsc*2^+/−^/poly I:C, genotype as between-subjects factor and cytokine as within-subjects factor: effect of genotype, *P* = 0.89; measure x genotype interaction, *P* = 0.34).

Taken together, these provisional data indicate that alterations in fetal brain cytokine abundance during MIA are less pronounced than placental changes and they suggest that cytokine responses during MIA are not substantially influenced by a heterozygous* Tsc2* mutation.

## 4. Discussion

In a previous study, we have found an interactive influence of* Tsc2* haploinsufficiency and gestational poly I:C on fetal development and adult behavioral traits in mice [24]: fewer *Tsc*2^+/−^ than WT pups were born to poly I:C-injected mothers, while the genotype ratio was balanced in vehicle-treated mothers, which suggested, together with the presence of fetal resorption, that gestational poly I:C triggered the preferential elimination of *Tsc*2^+/−^ fetuses* in utero*. Surviving poly I:C-exposed *Tsc*2^+/−^ animals, but not *Tsc*2^+/−^/saline and WT/poly I:C mice, showed abnormal social approach behavior when tested in adulthood [24]. Collectively, these data indicated that poly I:C-related gestational immune activation and* Tsc2* haploinsufficiency interact to perturb fetal development.

Gestational immune activation may, at least to some extent, interfere with normal fetal development because of adverse effects of cytokines on the developing organism [19, 22, 25–27]. TSC-related cell signaling regulates immunological and inflammatory processes [28, 29] and, therefore, it is conceivable that an exacerbated immune response in the *Tsc*2^+/−^  fetal brain to gestational poly I:C contributes to the interactive effects of* Tsc2* haploinsufficiency and maternal immune activation.

The present analysis of poly I:C-triggered cytokine responses suggests that *Tsc*2^+/−^ and WT fetal brains are exposed to qualitatively and quantitatively similar cytokine responses upon midgestational poly I:C administration. Cytokine antibody arrays revealed a pronounced increase in the abundance of specific cytokines in the placenta at 6.5 h after poly I:C, representing evidence for the mounted maternal immune activation triggered by poly I:C. Poly I:C caused a complex alteration in cytokine levels in the fetal brain that comprised an initial phase of decreased cytokine abundance (at 2 h after poly I:C) and a subsequent phase of modestly elevated tissue cytokine levels (at 6.5 h after poly I:C), which is consistent with prior data that show that the fetal brain cytokine response during maternal immune activation is a complex function of various parameters, such as time after poly I:C injection and gestational age [30]. Cytokine levels were similar in fetal *Tsc*2^+/−^ and WT brains, suggesting that genotype-dependent differences in cytokine exposure are unlikely to account for the interactive effects of* Tsc2* haploinsufficiency and gestational immune activation [24].

As TSC/mTOR signaling not only regulates immune responses, but also is a downstream signaling mediator of many cytokines and other factors induced by poly I:C, it remains to be explored if altered TSC-related cell signaling downstream of cytokines/other poly I:C-induced factors contributes to the genotype-dependent effects of poly I:C [24]. In addition, it is also possible that the disruptive effects of* Tsc2* haploinsufficiency and gestational immune activation converge on certain developmental processes ongoing at the time of poly I:C injection (E12.5). For instance, both gestational poly I:C and TSC gene mutations interfere with and disrupt neurogenesis, neuroblast migration, and proper cortical layering [31–33], which are important features of the developing CNS at E12.5. Accordingly, gestational immune activation and* Tsc2* haploinsufficiency may display synergistic effects on these processes and interactively lead to the phenotypic precipitation of otherwise subthreshold neurobiological changes.

## 5. Conclusion

The present study revealed a similar cytokine induction profile in* Tsc2* mutant and WT control brains in the context of a midgestational immune activation paradigm. These findings indicate that an exacerbated cytokine response in* Tsc2* mutants is unlikely to account for the interactive effects of* Tsc2 *haploinsufficiency and maternal immune activation identified in a previous study [24]. Future experiments will address if* Tsc2* haploinsufficiency and gestational immune activation may possibly interact by converging on cytokine-induced cell signaling and/or by jointly disrupting midgestational neurodevelopmental processes, such as neurogenesis and/or cell migration.
